# Unique sex chromosome systems in *Ellobius*: How do male XX chromosomes recombine and undergo pachytene chromatin inactivation?

**DOI:** 10.1038/srep29949

**Published:** 2016-07-18

**Authors:** Sergey Matveevsky, Irina Bakloushinskaya, Oxana Kolomiets

**Affiliations:** 1 Cytogenetics Laboratory, N.I. Vavilov Institute of General Genetics, Russian Academy of Sciences, Moscow 119991, Russia; 2 Evolutionary and Developmental Genetics Laboratory, N.K. Koltzov Institute of Developmental Biology, Russian Academy of Sciences, Moscow 119334, Russia

## Abstract

Most mammalian species have heteromorphic sex chromosomes in males, except for a few enigmatic groups such as the mole voles *Ellobius*, which do not have the Y chromosome and *Sry* gene. The ***Ellobius*** (XX ♀♂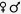) system of sex chromosomes has no analogues among other animals. The structure and meiotic behaviour of the two X chromosomes were investigated for males of the sibling species ***Ellobius** talpinus* and ***Ellobius** tancrei*. Their sex chromosomes, despite their identical G-structure, demonstrate short synaptic fragments and crossover-associated MLH1 foci in both telomeric regions only. The chromatin undergoes modifications in the meiotic sex chromosomes. SUMO-1 marks a small nucleolus-like body of the meiotic XX. ATR and ubiH2A are localized in the asynaptic area and the histone γH2AFX covers the entire XX bivalent. The distribution of some markers of chromatin inactivation differentiates sex chromosomes of mole voles from those of other mammals. Sex chromosomes of both studied species have identical recombination and meiotic inactivation patterns. In *Ellobius*, similar chromosome morphology masks the functional heteromorphism of the male sex chromosomes, which can be seen at meiosis.

The existence of two sexes and two types of gametes makes the exchange of genetic information possible, which is the main benefit of sexual reproduction. Meiosis is a key process in sexual reproduction; meiotic checkpoints purify unfit gametes and maintain the genetic integrity of the species.

The evolution of heteromorphic sex chromosomes started with the origin of the sex determination factor on the ancestor proto-Y chromosome^1^,^2^,^3^. In different mammalian groups, it was followed by numerous chromosomal rearrangements and the gradual loss of homologous regions, resulting in damage to the ability of such heteromorphic chromosome pairs to recombine^3^,^4^. Meiosis in the heterogametic sex demonstrates specific features, from complete synapsis to the lack of it.

In placental mammals, the gene *SRY* (Sex-determining Region Y) is a key factor that determines the formation of testes (the Testis Determining Factor, TDF) and is located on the Y chromosome^5^,^6^. In most placental mammals, the *X* and *Y* chromosomes contain short homologous regions (pseudoautosomal regions, PAR), which limit synapsis between them at the pachytene stage with the formation of short fragments of the synaptonemal complex (SC)^7^. There are no data for homologous regions of marsupial sex chromosomes^8^, but the possibility of a metatherian PAR is not excluded.

Sex chromosome systems in placental mammals that differ from the conventional XX/XY could be explained by translocations of autosomes and sex chromosomes or duplications of sex chromosomes. Usually, such atypical sex chromosomes are present in part of a population such as in the lemming *Myopus schisticolor*^9^, *Mus minutoides*^10^, and *Acomys*^11^. Nevertheless, in some species, such as in some howler monkeys, multiple sex chromosomes occur in all specimens^12^.

Some variants of duplications or nondisjunctions of sex chromosomes have been described for humans, for example XXY, XXXY, XXXXY, and XXYY^13^,^14^. Chromosomal changes cause different syndromes, including mental retardation and infertility. For example, de la Chapelle syndrome (46, XX, *SRY*-positive), which is classified as a testicular disorder of sex development, is diagnosed in 1 in 20,000 newborn boys^15^. In this syndrome, a fragment of the Y chromosome carrying the *SRY* gene is translocated to the X chromosome during paternal meiosis^16^. A similar case has been described for a tortoiseshell cat with 38, XX and the presence of the *SRY* gene due to Xp;Yp translocation^17^.

Occasionally, molecular studies have revealed the absence of the *SRY* gene in humans with male testicular disorder. Only 10% of the patients with XX are *SRY*-negative, and they usually demonstrate incomplete masculinization^18^. In nature, the existence of mammalian species with X chromosomes in both sexes and absence of the *SRY* gene in males seems incredible. Nonetheless, in at least two groups of rodents, that is the case: *Tokudaia* with X0^19^ and *Ellobius* with X0 and XX in both sexes^20^. These males are fertile; moreover, although *Tokudaia* are a rare species due to their small range of distribution, *Ellobius*, especially *E. talpinus* and *E. tancrei*, have broad distributions and occur in large numbers.

Five species of mole voles *Ellobius* are very unusual mammals with three types of sex chromosomes^21^. *E. fuscocapillus* (2n = 36, fundamental number (NF) or number of chromosomal arms is 60 for female and 59 for male) has a classical system of sex chromosomes, XY♂/XX♀. *E. lutescens* (2n = 17, NF = 34) has one sex chromosome, X in males and females. X chromosomes have a different morphology in *Ellobius*. In the *E. fuscocapillus* and *E. lutescens*, Xs are submetacentrics with different numbers of G-bands^21^. In the *E. fuscocapillus* pachytene XY chromosomes formed the PAR, which is typical for mammalian sex bivalents. In *E. lutescens*, the X univalent thickened during prophase I, becoming curved and variously shaped^22^. Three sibling species—the northern mole vole *E. talpinus* (2n = 54, NF = 54), the eastern mole vole *E. tancrei* (2n = 54-30, NF = 56) and the alai mole vole *E. alaicus* (2n = 52, NF = 56)—are characterized by two large acrocentric X chromosomes in females and males, which are equal in length and isomorphic for G- and C-bands^21^,^23^. The mechanisms of sex determination in *Ellobius* species remain enigmatic. There are no published data on whole-genome *Ellobius* studies; the identity of X chromosomes in males was proven by Zoo-FISH analysis^23^. Moreover, our previous study of female and male meiosis revealed functional distinctions for their X chromosomes^24^. We found that SCs that form between XX chromosomes were indistinguishable from autosomal SCs in oocytes—i.e., XX chromosomes underwent complete synapsis over the entire length of the SC bivalent. Male XX bivalent typically formed for the male mammal sex body, which moved to the periphery of the nucleus. Short fragments of SC were formed at telomeric regions of the bivalents only, and a wide zone of asynapsis between X and X chromosomes occurs in the central part of the SC bivalent. In these early studies, we found an electron-dense argyrophilic nucleolus-like body (Nlb) associated with one of the axial elements of the XX bivalent^22^.

The study of meiosis in males with XX is not a trivial task. The formation of chiasmas in PAR is essential for the proper segregation of the X and Y chromosomes during anaphase at the first meiotic division in the spermatocytes of most mammals. The evolution of heteromorphic sex chromosomes was synchronized with the origin of so-called MSCI (meiotic sex chromosome inactivation). MSCI is a multipurpose process, during which the chromatin of asynaptic regions of sex chromosomes undergoes transcriptional silencing^25^,^26^,^27^. Autosomes undergo silencing as well—this process has been named MSUC (meiotic silencing of unsynapsed chromatin). Upon the completion of autosome synapsis in meiotic prophase I, chromatin inactivation is replaced by its activation. In the case of the absence of synapsis up to the middle pachytene stage, as in heterozygotes with nonreciprocal aberrations for example, such asynaptic chromosomal regions are associated with sex bivalents and activate pachytene arrest, leading to the selection of spermatocytes^28^. A multistep process of chromatin reorganization underlies MSCI. First, the tumour suppressor protein BRCA1 (breast cancer 1) accumulates at non-synaptic areas of sex chromosomes, which starts the process of phosphokinase ATR (ataxia telangiectasia- and RAD3-related) recruitment^29^. BRCA1 and ATR proteins participate in further post-translation chromatin modification, including the phosphorylation of H2AFX^30^, ubiquitination of H2A^31^, sumoylation^32^ and others.

Mole voles offer the opportunity to explore these processes in the unique male XX sex chromosome system. We studied the recombination and pachytene chromatin inactivation of the male XX chromosomes, and the enigmatic nucleolus-like body associated with the SC of the sex bivalent in males of *E. talpinus* and *E. tancrei*. We also analysed the problem of sex chromosome divergence within the genus *Ellobius*, whose evolution has remained mysterious for several decades.

## Results

### Behaviour, synapsis and morphology of mole vole sex chromosomes

**Figure 1:**
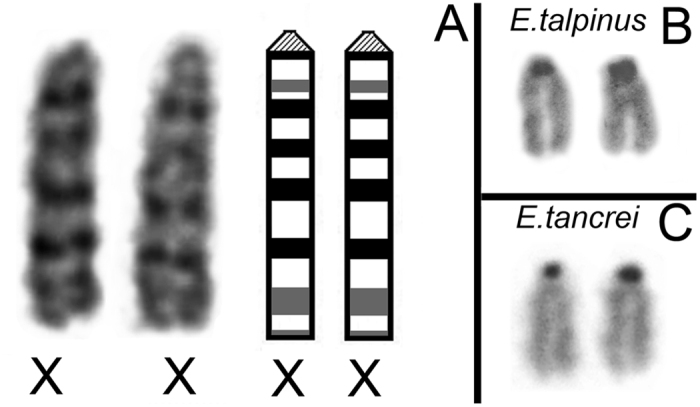
Male G- and C-band sex chromosomes of mole voles. (**A**) G-band sex chromosomes of *E. tancrei* (ХХ). For more details concerning G-band *E. tancrei*/*E. talpinus* sex chromosomes, see Bakloushinskaya *et al*.^23^ (**B**) C-band sex chromosomes of *E. talpinus*. (**C**) C-band sex chromosomes of *E. tancrei*.

We studied the synapsis features of the isomorphic XX chromosome (Fig. 1) by electron microscopy and immunocytochemistry in nuclei spreads of spermatocytes I.

**Figure 2:**
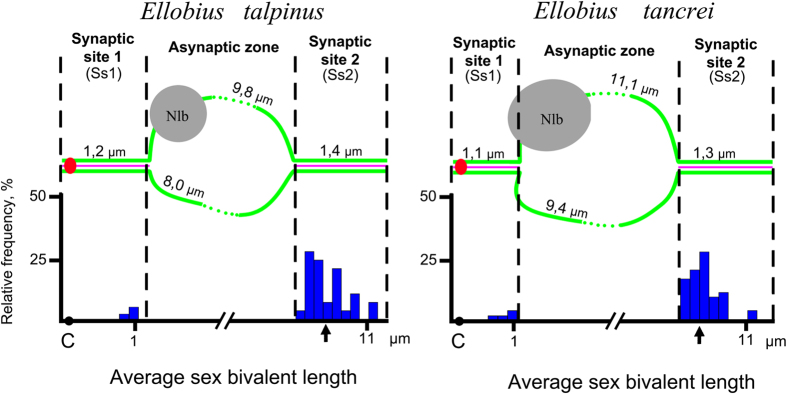
Morphometric parameters and the frequency of MLH1 foci in sex bivalents of mole voles. The *x*-axis shows the average length of sex bivalents in μm; the *y*-axis shows the relative frequency of MLH1 foci in each 10% of the length of sex bivalent. The arrows point to the central position of MLH1 in Ss2. Axial elements of the XX chromosomes are green, the central element of the SC in areas of synapsis is pink, the centromere is red, and Nlbs are grey. The frequency of MLH1 is presented graphically as blue bars.

**Figure 3:**
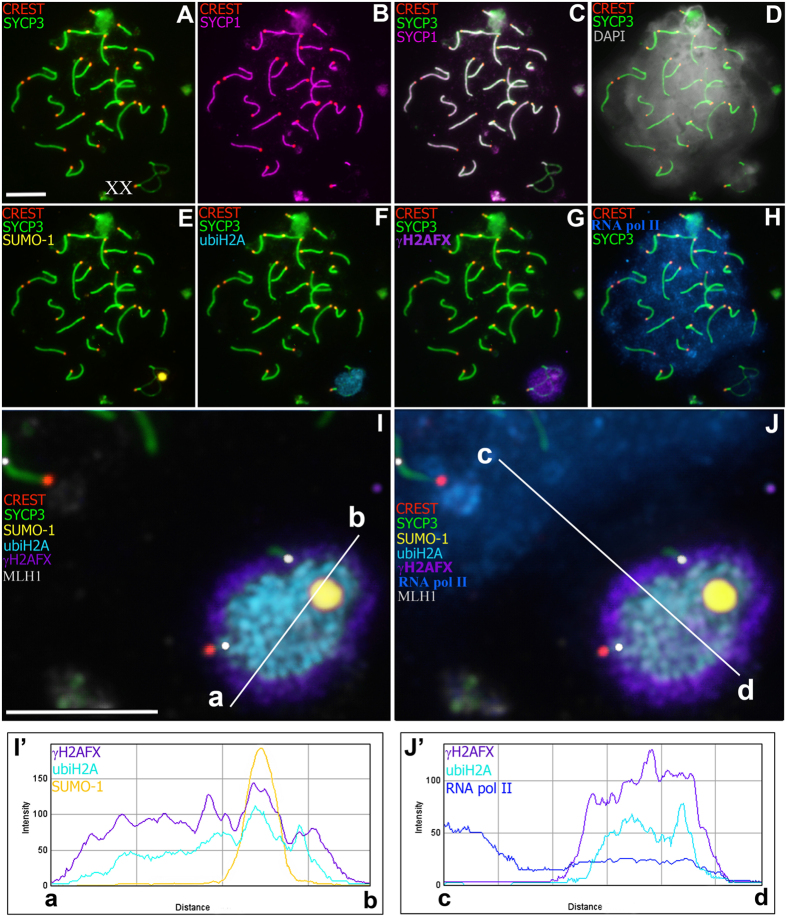
*Ellobius talpinus* pachytene spermatocyte after multiple sequential immunostaining assays. Bar = 10 μm. Localization of SYCP3 (green), SYCP1 (magenta), CREST (red), SUMO-1 (yellow), ubiH2A (cyan), RNA pol II (blue), γH2AFX (violet), MLH1 (grey) and DAPI (grey). (**A–H**) The same spermatocyte was stained with different antibodies. (**I,J**) Sex (XX) bivalent (from **A–H**). MLH1-signals were observed in telomeric synapsis, proteins of inactivation were localized at the asynaptic zone. SUMO-1 was located within the nucleolus-like body (Nlb) (arrow). Intense RNA pol II signal was detected in the main part of spermatocytes; within the sex bivalent, it was less intense. Co-localisation of SUMO-1, ubiH2A, RNA pol II, and γH2AFX is shown in graphs (**a–d**) (see **I**’ and **J’**).

**Figure 4:**
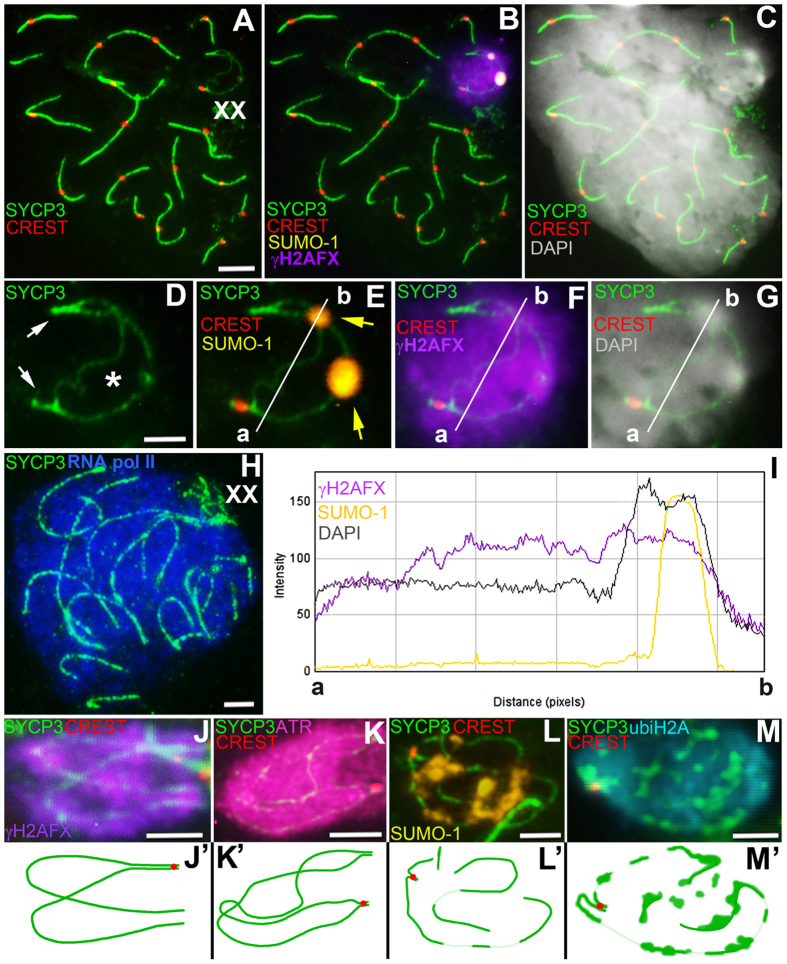
Pachytene spermatocytes and male sex (XX) chromosomes of *Ellobius tancrei*. Bar (**A–C, H**) = 5 μm; Bar (**D–G**, **J–M**) = 2 μm. Axial elements were identified using anti-SYCP3 antibodies (green), and anti-CREST for kinetochores (red). (**A–C**) Spermatocyte at the pachytene stage. The sex bivalent was displaced to the periphery of the nucleus. (**D–G**) The sex (XX) bivalent (from Figs **A–C**) has two regions of synapsis (white arrows) and a wide asynaptic area (asterisk) (see **D**). SUMO-1 (yellow) immunostained Nlb (yellow arrows) in the form of two large round spots (see **E**). γH2AFX (violet) surrounded the entire sex bivalent (**F**). DAPI-signal (grey) surrounded the sex bivalent (see **C**), with the more intense signal in the Nlb (see **G**). (**H**) The RNA pol II signal was intense in the main part of the spermatocyte. RNA pol II had a weak staining signal within the sex bivalent. (**I**) Co-localization of SUMO-1, γH2AFX, and DAPI is shown in graph (**a,b**) (see **E–G**). (**J**) The sex bivalent at the early pachytene stage was intensely γH2AFX stained (violet) (scheme **J’**). (**K**) The asynaptic area of the sex bivalent was intensely ATR stained at the mid pachytene stage (scheme **K’**). (**L**) The sex bivalent rolled up into a tangle at the late pachytene stage (scheme **L’**). Axial elements of the XX bivalent had SYCP3 gaps, which were marked with SUMO-1 (yellow). (**M**) The sex bivalent underwent reorganization at the early diplotene stage (scheme **M’**). At the same time, the gaps in the axial elements are interspersed with clusters of SYCP3 along the chromosomes, including the area near the centromere, and were surrounded by ubiH2A (cyan).

The sex bivalent was located among the autosomes at the zygotene stage. Later, the XX bivalent was gradually displaced to the periphery of the nucleus, forming an electron-dense sex body by the end of the mid pachytene stage as is typical for mammalian males. The XX sex chromosomes of *E. talpinus* and *E. tancrei* males underwent a partial synapsis between the short telomeric regions in meiotic prophase I. Thus, their XX sex chromosomes form a closed bivalent with a central asynaptic zone (Figs 2, 3A, 4A,D and S4A,E,I). At synaptic sites (Ss), SYCP1 (synaptonemal complex protein 1) immunostaining revealed a central element, indicating the formation of a typical SC (Fig. 3B,C).

At the middle pachytene stage, axial elements twisted together and acquired a figure-eight configuration (Figs 3A and 4A). By the end of pachytene, the sex bivalent curled up into a tangle (Figs 4L, S1I and S2). SYCP3, the main protein of the axial and lateral elements of the SC, was gradually removed from the axial elements at early diplotene (Figs 4M and S2), wherein gaps in the axial elements alternated with SYCP3 clusters along the chromosomes and near the centromeres (Fig. 4M). At the late diplotene stage, some nuclei could be observed in which only the sex body was stained with anti-SYCP3 antibodies because autosomal axial elements no longer contained SYCP3.

The average length of *E. talpinus* sex bivalents was 11.6 ± 2 μm (range: 8.2 to 16.3 μm), while the average length of *E. tancrei* sex bivalents was 12.6 ± 2.5 μm (range: 8.3 to 17.7 μm). The length of the asynaptic regions of the XX chromosomes in *E. tancrei* and *E. talpinus* varied (range: 5.5 to 14.4 μm for *E. talpinus* and 5.2 to 16.4 μm for *E. tancrei*). The length of the synaptic regions also varied, in general becoming shorter towards the end of pachytene SC fragments (Fig. S1I).

Centromeres were identified by immunodetection with the CREST antibody. We have designated the pericentromeric SC region between XX chromosomes as Ss1, and the distal part of the SC as Ss2 in both species (Table 1, Figs 2, 3A and 4A). Centromeres were located at 0.2 and 0.3 μm from the telomere in *E. talpinus* and *E. tancrei*, respectively (Table 1, Fig. 2). A comparison of the SC fragment lengths (Ss1 and Ss2) revealed no significant differences between these fragments in *E. tancrei* and *E. talpinus* (*P* = 0.5 and *P* = 0.3, respectively). Thus, the length of the *E. tancrei* axial elements was greater than those of *E. talpinus* due to their longer asynaptic regions. It should be emphasized that the distal part of the SC (Ss2) was not detected by immunostaining with antibodies against SYCP3 in the sex bivalent structure at the pachytene stage in 24% of *E. talpinus* and 27% of *E. tancrei* nuclei; however in electron micrographs, using AgNO_3_ for contrast, this SC fragment was usually visible but disappeared later at the diplotene stage (Fig. S2).

In our previous electron microscopy study of sex bivalent structures we revealed one or two nucleolus-like bodies (Nlb) in *E. tancrei* and *E. talpinus*^28^ (Figs 2 and S2). The nature of the Nlbs has not been established. The nucleolar nature of these structures has been proposed based on their high argyrophility, high density and shape. Nlbs vary in size and shape, and the patterns of their structural organization are described below.

### MLH1 in mole vole sex (XX) chromosomes structure

Antibodies against the mismatch repair protein MLH1 (mutL homolog 1) detected crossing over sites in 94 *E. talpinus* and 114 *E. tancrei* nuclei of pachytene spermatocytes. We could identify the MLH1 signals in XX bivalent structures for 46% of *E. talpinus* and 65% of *E. tancrei* nuclei (Table 1, Fig. 2). One or two MLH1 foci were detected at sex bivalents in both species. Simultaneously, one signal for each of the synaptic sites (Ss1 and Ss2) was detected at two *E. talpinus* and four *E. tancrei* sex bivalents (Fig. 3J).

The average distance between the telomere and MLH1 focus was 1.3 ± 0.7 in Ss1, and 0.93 ± 0.5 in Ss2 for *E. talpinus* nuclei; for *E. tancrei* nuclei, the average distances were 1.04 ± 0.1 in Ss1, and 0.96 ± 0.5 in Ss2 (Table 1, Fig. 2). The average distance between the MLH1 focus and telomeres was 74% of the length of the Ss2 for *E. talpinus* and 69% for *E. tancrei* (Fig. 2, highlighted by an arrow). Significant differences in the position of the late recombination nodules in Ss2 for these species were not found (*P* = 0.08).

However, in *E. talpinus* nuclei, in the short sections of Ss1 and Ss2 (up to 10% of the length of the zones) flanking the asynaptic zone, MLH1 foci were not identified or detected in only a few cases. Perhaps this difference in the distribution of late recombination nodules in the structure of the XX bivalent between two sibling species arose due to the lower number of identified MLH1 signals in the *E. talpinus* sex bivalents.

### Chromatin remodeling in the mole voles male XX body

**Figure 5:**
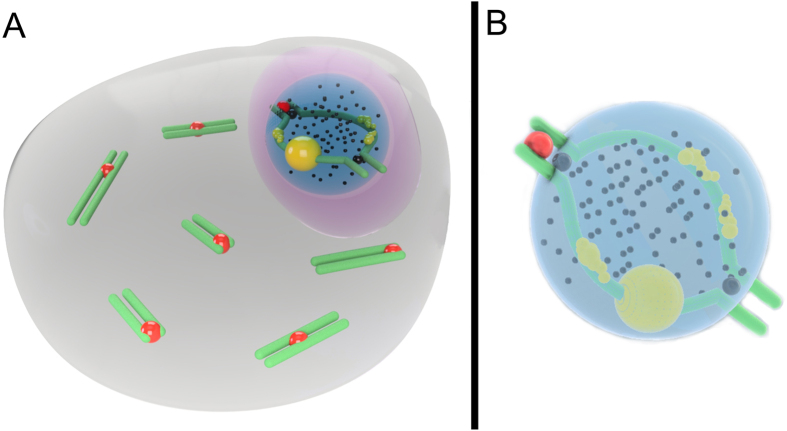
Schematic illustration of male Ellobius MSCI. A pachytene spermatocyte (**A**) and a sex (XX) bivalent (**B**) of a mole vole are shown. The sex (XX) chromosomes form a sex body on the periphery of the nucleus. The chromatin of the sex body undergoes reorganization. MSCI markers have different distributions: SUMO-1 (yellow), ATR (black dots), ubiH2A (blue), H2AFX (violet). SUMO-1 marks a small nucleolus-like body of the meiotic XX. ATR and ubiH2A are localized in the asynaptic area of the sex bivalent. γH2AFX covers the entire XX bivalent. The approximate number of autosomal SCs is shown. MLH1 signals are shown only for the sex chromosomes (black balls). The red balls indicate centromeres.

At the early pachytene stage, when sex bivalents have not yet become tangled, chromatin was grouped around the axis elements of the XX chromosomes (Fig. 4J). The histone γH2AFX was not detected at telomeric synaptic sites of sex bivalents (Ss1 and Ss2) (Table S1, Fig. 3G,J). In middle pachytene stage spermatocytes of both species, the distribution of histone γH2AFX in the structure of the sex body coincided with XX chromosome chromatin when counterstained with DAPI (Figs 5В and S1M–P). In *E. tancrei* nuclei, the ATR protein was detected in the pachytene chromatin, which surrounded the sex bivalent, with more intense fluorescence at the asynapsis zone (Fig. 4K and S1A–C).

SUMO-1 appears in the structure of sex bivalents of mole voles closer to the middle pachytene. The SUMO-1 signal was detected intensively in the Nlb area of XX bivalents in both species (Figs 3E, 4E and S1E–H). When sex bivalents curled into a tangle at the late pachytene stage, SUMO-1 was often detected in areas of axial elements free of SYCP3 (Fig. 4L).

ubiH2A was detected in the XX asynaptic zone (Fig. 3F). If the sex bivalent rolled into a tangle—i.e., synaptic regions were close to each other, but not on opposite sides (in the post pachytene stages)—ubiH2A was detected within the entire XX bivalent (Figs 4M and S1I–L).

Intensity correlation analysis (ICA) and analysis of the fluorescence intensity profile (FIP) allowed the assessment the degree of MSCI protein co-localization (Figs 3I’-J’, 4I and S3). Thus, the degree of co-localization (m ± SD) is high for γH2AFX and ubiH2A (Pearson correlation coefficients: *r*_p_ = 0.90 ± 0.10; Overlap correlation coefficients: *r* = 0.93 ± 0.06 for *E. talpinus* and *r*_p_ = 0.82 ± 0.16, *r* = 0.90 ± 0.09 for *E. tancrei*; *P* = 0.45). Regarding the fluorescence-intensity profiles, the ubiH2A-signal path (Fig. 3I’–J’) was similar to the γH2AFX-signal path, but smaller in the width of coverage—as a rule, it covered only the asynaptic zone. γH2AFX has a wide distribution in the XX bivalent. The high degree of ubiH2A and γH2AFX co-localization within the sex body was noted previously^31^. γH2AFX/ATR and ubiH2A/ATR are highly colocalized (*r*_p_ = 0.70 ± 0.19, *r* = 0.88 ± 0.06 and *r*_p_ = 0.72 ± 0.08, *r* = 0.89 ± 0.05, respectively; only for *E. tancrei*) (Fig. S4). A lesser degree of co-localization was revealed for the γH2AFX/SUMO-1 (*r*_p_ = 0.45 ± 0.10, *r* = 0.59 ± 0.11 for *E. talpinus* and *r*_p_ = 0.48 ± 0.14, *r* = 0.64 ± 0.13 for *E. tancrei*; *P* = 0.17) and ubiH2A/SUMO-1 (*r*_p_ = 0.63 ± 0.17, *r* = 0.69 ± 0.14 for *E. talpinus* and *r*_p_ = 0.60 ± 0.13, *r* = 0.73 ± 0.17 for *E. tancrei*; *P* = 0.53) combinations (Figs S3 and S4). It should be emphasized that in the case of multiple immunostaining assays, the bright signal of the fluorescent label conjugated with antibodies to SUMO-1 and detected in Nlb did not disappear during photobleaching and thorough washing with PBS, although the “burning” of fluorescence was easily managed for other antibodies. Because anti-SUMO-1 antibodies were used earlier than ubiH2A and γH2AFX, we observed a characteristic peak in the paths of the ubiH2A-, γH2AFX-signal fluorescence-intensity profiles (Fig. 3I’). Thus, it was necessary to duplicate all experiments using one of the MSCI proteins (Fig. S1), eliminating the possibility of the manifestation of signals from earlier rounds of immunostaining.

### RNA pol II in mole vole XX bodies

RNA pol II was either not detected in the chromatin of sex bivalents by immunostaining or its fluorescence intensity was significantly lower than that in autosomal chromatin for both species of mole voles (Figs 3H and 4H), a finding that is confirmed in the graph **cd** (Fig. 3J,J’). The intensity of the RNA pol II signal was analysed via the **cd** path, drawn from the main part of spermatocytes through the sex bivalent. The signal was less than half as intense in the area surrounding the XX bivalent than in other parts of the pachytene nuclei.

### DAPI staining of mole vole meiotic sex chromosomes

The DAPI signal was identified in all mole vole spermatocytes. The most intensive DAPI staining was observed for the Nlb of sex bivalents in *E. talpinus* and *E. tancrei* (Figs 3D, 4C,G and S1H,L). When the axis of the sex bivalent formed a tangle at the late pachytene stage, the Nlb was sometimes absent (Fig. S1P).

## Discussion

Analysing the distribution of crossover-associated MLH1 signal along mole vole male sex chromosomes, we found a high frequency of foci close to the asynaptic zone and a lower frequency of signals in telomeric areas. At a distance of 65–75% of the length of Ss1 (counting from the telomere-centromere), MLH1 signals were absent in sex bivalents of both species. A decrease or absence of recombination near centromeres has been previously described for different species: muntjac^33^, elephant shrew^34^,^35^, mouse, humans^36^,^37^ and others. It is assumed that the suppression of recombination in the centromere area (centromere effect) is due to the presence of centromeric heterochromatin^38^. Despite the small amount of C-heterochromatin localized in the sex chromosomes of the studied mole voles (Fig. 1B,C), it was still able to influence the distribution of MLH1. The centromeric effect is likely to be significantly linked to the lower rate of recombination in Ss1 than in Ss2 for both species. In Ss2, the recombination peak was identified outside of the telomeric zone. Apparently, that may be due to the mechanical containment of synaptic sites, as if the asynapsis area is severely restricted on both sides.

The low number of sex (XX) bivalents with MLH1 foci (46% *E. talpinus*, 65% *E. tancrei*) is likely due to two reasons. First, a heavy and compact protein envelope causing inactivation and covering the sex chromosomes at prophase I could prevent the penetration of anti-MLH1 antibodies. Second, SYCP3 could be removed from Ss2 early in the pachytene stage. In this case, the MLH1 could be detected at the sex bivalent for a short time. The number of sex bivalents with MLH1 varies not only among males of different species but also conspecific males. The percentage of males having sex bivalents with MLH1 was 17,47-45,8% in bulls, 20,56% in barbary sheep^39^,^40^, 29–30% in wildebeest and antelopes^39^,^41^, 60.6% in muntjacs^33^, 61–74% in different breeds of pigs^42^, 73% in humans^43^, and 80% in horses^44^. The recombination patterns of the sex chromosomes in both species of mole voles were similar and appeared to be different from those of other mammalian species.

The presence of two recombination peaks, one at each synaptic site of the sex chromosomes suggests that the two ancestral X chromosomes were structurally isomorphic. We hypothesize that later they underwent a secondary heteromorphization and became functionally different in the central region. The evolutionary assumption, based on ancestral male mole voles having the XY system, is that the loss of the Y chromosome was the first event, and doubling of the X chromosome occurred thereafter.

During MSCI, sex chromosomes are evicted to the periphery of the meiotic nucleus and form a so-called sex body^25^. A sex body is a morphological structure that marks the MSCI. It should be noted that the failure of sex body formation in spermatocytes might correlate with infertility in heterozygotes for chromosomal rearrangements^45^. Epigenetic chromatin modifications of sex bodies have been conducted using antibodies against proteins involved in the MSCI process.

ATR is involved in the formation of the sex body at prophase I^29^,^46^. Normally, ATR starts to be eliminated at middle zygotene upon the development of synapsis between the chromosomes and disappears later; at pachytene, ATR is only detected at asynaptic regions of the sex chromosomes^47^,^48^. In *E. tancrei*, ATR was localized in unsynapsed chromatin of the central part of the pachytene XX bivalent. We observed a similar situation in the spermatocytes of interspecific hybrids of *E. tancrei* and *E. talpinus* (unpublished data). Unfortunately, we have not analysed the distribution of ATR foci in *E. talpinus*, but we expect similar results.

Phosphorylation of H2AFX is an ATR-dependent process, and phosphorylated histone γH2AFX appears twice during prophase I. The first wave of phosphorylation comes at leptotene, when DSB development occurs, and the second wave starts at pachytene coincident with sex body formation^30^. The phosphorylation of H2AFX is considered to be an important event for the initiation of MSCI. Mouse null mutants of γH2AFX demonstrated deviations in sex body formation^49^. In mutants of SYCP1 and BRCA1, an aberrant pattern of γH2AFX and ATR localization was observed^48^,^50^. In mole vole spermatocytes, we observed γH2AFX in the sex body from early pachytene. At the transition from the early to the middle pachytene stages, γH2AFX was localized only at the XX bivalent asynaptic zone. At the late pachytene stage, γH2AFX appeared throughout the entire sex bivalent. Thus, it is possible that the chromatin in the synaptic sites inside the XX body was also modified.

SUMO (small ubiquitin-related modifier) group proteins are involved in various cellular processes, including DNA replication, recombination, repair and formation of heterochromatin-specific nuclear areas^32^. SUMO-1 plays an important role in SC and sex body formation, as well as in maintaining the structure of chromatin^51^,^52^. In mouse and rat XY bodies, SUMO-1 is located along the asynaptic sites of the sex chromosomes^51^,^53^. Therefore, we were surprised to discover that SUMO-1 localized within the Nlb at the XX bivalent. Maximum staining for SUMO-1 at the sex body of *E. talpinus* and *E. tancrei* occurred at the middle pachytene stage. During the transition from pachytene to diplotene, when SYCP3 protein is partially eliminated from the axial elements, it is possible to observe SUMO-1 localization. This was shown for *E. tancrei* only (Fig. 5L). Until now, the nature of the AgNO3-positive Nlb was not clear. Previously, we could not immunodetect nucleolar RNA-binding protein nucleophosmin/B23 in mole vole sex bodies^54^. In this study, we demonstrate that the Nlbs are SUMO-1- and DAPI-positive sites and seem to have a chromatin nature.

The mono-ubiquitinated histones H2A (ubiH2A) and H2B play an important role in nuclear processes, including transcription, DNA repair and the maintenance of chromatin structure^55^. In pachytene spermatocytes of mice, rats and humans, ubiH2A was localized within the asynaptic chromatin of the XY bivalent^31^,^53^. In mole voles, we observed a similar picture, and an intense signal of ubiH2A was localized in the asynaptic area of the XX bivalent. Assessing the co-localization of ubiH2A with γH2AFX, we showed that ubiH2A was distributed in a smaller area of the XX sex bivalent. As suggested by Baarends *et al*.^31^, ubiH2A is not responsible for the formation of silent chromatin but is only involved in maintaining it in its inactive form. Apparently, the same is true for the mole vole sex body.

RNA pol II, a marker of chromatin transcriptional activity, is highly concentrated in autosomal chromatin but much less concentrated in sex body chromatin^48^,^56^. There is also a link between low levels of RNA pol II and high levels of γH2AFX and ATR in mouse sex bivalent^35^. We observed a similar link between the RNA pol II and MSCI proteins in the mole vole sex bivalent.

The Nlb was clearly revealed in the structure of one of the XX bivalent axes early in the zygotene stage. As seen from the above results, asynchronous chromatin inactivation occurs later in asynaptic parts of the XX chromosomes. The XX chromosome isomorphism denotes their homology; additionally, we assume that asynapsis may be caused not by a lack of homology between isomorphic XX but by their early inactivation and specific heterochromatization of some regions of at least one of the X chromosomes. In other words, we assume that early asynchronous epigenetic chromatin changes lead to absence of synapsis between isomorphic XX chromosomes. Obviously, whole-genome sequencing will resolve this issue.

We propose a model of pachytene chromatin remodeling in the sex body for the two mole vole species by the identification of MSCI markers (Fig. 5). Obviously, the processes of MSCI, as well as the patterns of recombination, are similar in *E. talpinus* and *E. tancrei*. Thus, isomorphic G-band XX sex chromosomes of mole vole males behave normally in prophase I of meiosis. As in all male mammals, they formed areas of synapsis and asynapsis, shifted to the periphery of pachytene nuclei, and underwent MSCI.

The main characteristic of a Y chromosome is that it carries a testis-determining factor, such as *Sry*, in placental mammals. The *Sry* gene encodes a transcription factor that up-regulates the *Sox9* gene via binding of the testis-specific enhancer (TESCO)^57^. Recent studies on *Sox9* gene regulation have demonstrated unusual results common between four *Ellobius* species, with or without Y chromosome and *Sry*^58^. All of the studied species carry a 14-bp deletion (Δ14) in the highly conserved module of the *Sox9* cis-regulatory element, TESCO. It is possible that the deletion Δ14 led to up-regulation of the *Sox9* gene by increased activity of TESCO. It is known that XX transgenic mice over-expressing *Sox9* develop as infertile males^59^, but XX males and females of *E. talpinus* and *E. tancrei* are fertile. Moreover, in the *Sry*-positive species *E. fuscocapillus*, a point mutation (A to G change) in the TESCO module ECRiii, might abolish *Sry* binding to its target site R5^58^. In that case, the presence of the *Sry* gene in the *E. fuscocapillus* genome is redundant due to loss of its functional target binding site.

In similar model species of *Tokudaia*, TESCO has lost its enhancer functions for the *Sox9* gene in XO species without the *Sry* gene and in XY species with multiple *Sry* gene copies^60^ caused by different mutations than in *Ellobius*, but with similar effects. Apparently, the loss of the *Sry* gene triggers an alternative, still unknown gene for sex determination. Such changes in enhancer structure could be crucial for genome stability and provoke the loss of the *Sry* gene and the Y chromosome in *Ellobius* and *Tokudaia*.

The manifestation of typical XY sex bivalent meiotic patterns in case of XX male sex bivalents in *E. talpinus* and *E. tancrei* may be a first step in the evolution from isomorphic sex chromosomes into heteromorphic ones. In *Ellobius*, with XX chromosomes in both sexes, chromosome morphology masked the functional heteromorphism of the sex chromosomes, which can be observed only at meiosis. *Ellobius* seems to be a natural model for the study of the evolution of sex chromosomes and molecular mechanisms of sex determination in mammals.

## Materials and Methods

### Animals

We studied two males of *E. talpinus*, and six males of *E. tancrei*: one 2n = 54, one 2n = 50 and four 2n = 34. We used animals from a long-term stock, which has been maintained at the Koltzov Institute of Developmental Biology; *E. talpinus* were originated from the Orenburg District, Russia, and all forms of *E. tancrei* originated from Pamiro-Alay, Tajikistan. The animals were maintained under comfortable conditions, enabling them to reproduce. All of the specimens were karyotyped. The animals were treated according to established international protocols, such as the Guidelines for Humane Endpoints for Animals Used in Biomedical Research. All of the experimental protocols were approved by the Ethics Committees for Animal Research of the Vavilov Institute of General Genetics and the Koltzov Institute of Developmental Biology in accordance with the Regulations for Laboratory Practice in Russian Federation.

### Meiotic spread preparations

The suspension of spermatocytes was placed in a centrifuge tube and washed by centrifugation with 10 ml of Eagle’s medium, 2–3 times for 10 minutes at 1500 rpm. Thereafter, the tube with cells was placed on ice. Synaptonemal complex preparations were made and fixed using the technique previously described^22^,^24^.

### Electron microscopy study of SCs

Slides for a subsequent electron-microscopic study were covered with Falcon plastic and stained with a 50% AgNO_3_ solution in a humid chamber for 3 h at 56 °C, then washed 4 times in distilled water and air dried. Stained slides were examined under a light microscope to select suitably spread cells. Once selected, Falcon plastic circles were cut out with a diamond knife, transferred onto grids, and examined at a JEM 100B electron microscope.

### Antibody and immunostaining procedure

Poly-L-lysine-coated slides were used for all immunofluorescence studies. The slides were washed with phosphate-buffered saline (PBS) and incubated overnight at 4 °C with primary antibodies diluted in antibody dilution buffer (ADB: 3% bovine serum albumin—BSA, 0.05% Triton X-100 in PBS). The primary antibodies used were rabbit polyclonal anti-SYCP1 (1:500, Abcam, #15090), rabbit polyclonal anti-SYCP3 (1:500–1:1000, Abcam, #15093), mouse monoclonal anti-ATR (1:200, Abcam, #54793), human anticentromere antibody CREST (1:500, Fitzgerald, #90C), mouse monoclonal anti-MLH1 (1:50, Abcam, #59756), mouse monoclonal anti-SUMO-1 (1:250, Zymed, #33-2400), mouse monoclonal anti-ubiquityl histone H2A (1:400, Millipore, #05-678), mouse anti-RNA polymerase II (RNA pol II) (1:1000, Abcam, #5408) and mouse anti-phospho-histone H2AX (1:1000, Abcam, #22551). After washing, we used the corresponding secondary antibodies diluted in PBS: FITC-conjugated bovine anti-rabbit IgG (1:1000, Santa Cruz Biotechnology), goat anti-rabbit Alexa Fluore 488 (1:500, Invitrogen), FITC-conjugated horse anti-mouse IgG (1:500, Vector Lab.), Rodamine-conjugated chicken anti-rabbit IgG (1:400, Santa Cruz Biotechnology), goat anti-human Alexa Fluore 546 (1:500, Invitrogen), and goat anti-mouse Alexa Fluore 546 (1:200, 1:1000, Invitrogen). Immunostaining was carried out sequentially in several rounds. The first round included the simultaneous use of two primary antibodies, SYCP1+MLH1, and then washing in PBS. The slides were subsequently processed with the corresponding secondary antibodies, were washed in PBS, and then were mounted in Vectashield with 4′,6-diamidino-2-phenylindole (DAPI) (Vector Laboratories). The slides were examined using an Axioimager D1 microscope (Carl Zeiss, Jena, Germany) equipped with an Axiocam HRm CCD camera (Carl Zeiss) and image-processing AxioVision Release 4.6.3. software (Carl Zeiss). All of the preparations were counterstained with DAPI. Each cell was photographed three times to document the results of SYCP1, MLH1, DAPI staining. The coordinates of each photographed nucleus were recorded. Next, we “burned” off the fluorochromes that were used during the first round. The slides were kept for several minutes under a halogen lamp for full or partial elimination (“burning”) of fluorochrome fluorescence. The slides were then washed in PBS (5–7 times for 6–8 minutes each). We conducted the second round in the same manner as for the first round, this time using antibodies against SYCP3 + CREST. In the third round we used antibodies against ATR (or SUMO-1). In the fourth round we used antibodies against ubiH2A, in the fifth antibodies against RNA pol II were used. In the sixth round, we used antibodies against γH2AX (also known as γH2AFX). After each round, the slides were washed in PBS and mounted in Vectashield with DAPI (Vector Laboratories). The number of rounds varied. The use of multi-round immunostaining allowed us to identify up to eight antigens in the same nucleus.

### Control

As a control for the co-localization of proteins and to avoid errors when using the same host antibodies, control staining was performed in parallel on other slides, which were treated with two or three antibodies (e.g., only antibodies against SYCP3-SUMO-1 or against SYCP3-ubiH2A or against SYCP3-γH2AFX).

### Co-localization and intensity correlation analysis (ICA) or the «dye-overlay» method

An assessment of the co-localization of various proteins in the same part of the cell was made by overlaying photographs taken separately using different channels. ICA was performed according to Reitan *et al*.^61^. In the analysis of the co-localization of proteins involved in the process of MSCI, we considered several characteristics of the distribution of proteins, using scatter plots, Pearson correlation coefficients (*r*_p_), overlap correlation coefficients (*r*) and individual fluorescence intensities, which were obtained using the plug-Intensity Correlation analysis^62^, and ImageJ version 1.45o, available in the public domain^63^,^64^. ImageJ converts primary (raw) microscopic images in an 8-bit format and analyses the degree of co-localization. *r*_p_ and *r* allow us to estimate the correlation between different fluorescence intensities. *r*_p_ ranged from −1 (in the case of strict negative correlation) to +1 (strictly positive correlation). It is worth noting that various software plugins for co-localization can calculate differing values for *r*_p_^65^; thus it was important to use the same plugin for analogous assessments. In analysing scatter plots, overlaying green and red signal resulted in a yellow signal (see the scatter plots in Fig. S3). The more yellow in the scatter plot was, the higher the level of overlap. The width of the distribution of the yellow signals in scatter plots corresponded to the degree of co-localization of the compared fluorescence signals: the wider the distribution of the signal was, the higher the level of overlap of the two channels in the study area would be. *P* value was calculated for *r* (see the Results section).

### Fluorescence-intensity profile (FIP)

To assess the degree of co-localization for several proteins along a given line, we made fluorescence-intensity profiles (FIPs) using the RGB profiler function in the ImageJ program according to previously published methods^66^,^67^.

### Statistical analysis

All of the data are shown as the mean values ± SD. Student’s *t*-test was performed to determine significant differences in the data. All statistical analyses were conducted using GraphPad Prism Version 5.0 (GraphPad Software, CA, USA). *P* value < 0.05 was considered to be statistically significant.

## Additional Information

**How to cite this article**: Matveevsky, S. *et al*. Unique sex chromosome systems in *Ellobius*: How do male XX chromosomes recombine and undergo pachytene chromatin inactivation? *Sci. Rep.* **6**, 29949; doi: 10.1038/srep29949 (2016).

## Supplementary Material

Supplementary Information

## Figures and Tables

**Table 1:** Morphometrics of *E. talpinus* and *E. tancrei* pachytene sex bivalents.

Species	No. of spermatocytes analysed (all stages)			Average length of pachytene sex bivalent, μm (mean ± SD)					Sex bivalent with MLH1
	N (P)	IF	EM	Ss 1	Asynaptic zone (AZ)		Ss 2	Tel.–Cen. distance	
					AE 1	AE 2			
*E. talpinus*	171 (129)	154	17	1.2 ± 0.7	9.8 ± 2.6	8.0 ± 2.9	1.4 ± 0.8	0.2 ± 0.09	46%
*E. tancrei*	351 (232)	317	34*	1.1 ± 0.5	11.1 ± 3.1	9.4 ± 2.5	1.3 ± 0.5	0.3 ± 0.1	65%

P–pachytene nuclei, IF–immunofluorescence, EM–electron microscopic data. AE1 and AE2–axial elements 1 and 2. AE1 has the nucleolus-like body and is longer than AE2. Ss1 and Ss2–synaptic sites 1 and 2. Tel.–telomere. Cen.–centromere. *-Since 1983, more than 1,000 spermatocytes were analysed by electron microscopy; here are the data on the investigated animals in this study.
