# Supplementary material for: Unique sex chromosome systems in Ellobius: How do male XX chromosomes recombine and undergo pachytene chromatin inactivation?
Source: Sci Rep. 2016 Jul 18;6:29949. doi: 10.1038/srep29949 (PMC4947958; doi:10.1038/srep29949)
Supplement: Supplementary Information [file srep29949-s1.doc]

Supplementary materials

**Unique sex chromosome systems in Ellobius: How do male XX chromosomes recombine and undergo pachytene chromatin inactivation?**

Sergey Matveevsky^1^*, Irina Bakloushinskaya^2^, Oxana Kolomiets^1^

^1^Cytogenetics Laboratory, N.I. Vavilov Institute of General Genetics, Russian Academy of Sciences, Moscow 119991, Russia.

^2^Genetics Laboratory, N.K. Koltzov Institute of Developmental Biology, Russian Academy of Sciences, Moscow 119334, Russia

* [sergey8585@mail.ru](mailto:sergey8585@mail.ru)

| **Antibodies** | ***E. talpinus*** | ***E. tancrei*** |
| --- | --- | --- |
| γH2AFX | 87 | 156 |
| ubiH2A | 63 | 115 |
| SUMO-1 | 52 | 93 |
| ATR | - | 45 |
| RNA pol II | 41 | 79 |
| MLH1 | 94 | 114 |
| **Total (IF)** | **154** | **317** |

Table S1. Number of analysed cells. IF - immunofluorescence. The calculations include the cells after multiple sequential immunostaining assays and cells after staining with two antibodies simultaneously (SYCP3 + antibody to one of MSCI proteins).


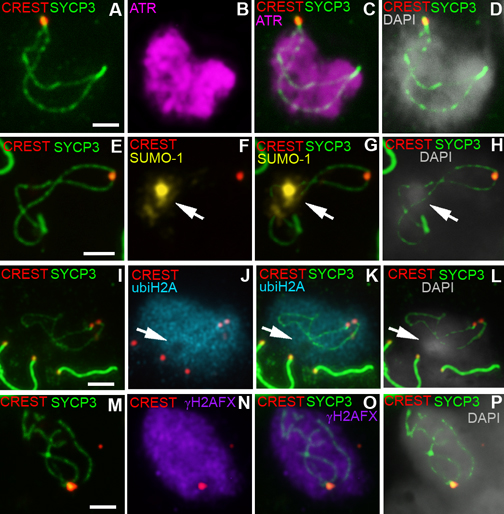


Figure S1: Pachytene male (XX) sex chromosomes of *Ellobius tancrei* (A-D) and *Ellobius talpinus* (E-P)**.** Bar = 2µm. Immunostaining with antibodies anti-ATR (magenta), anti-SYCP3 (green), anti-CREST (red), anti-SUMO-1 (yellow), anti-ubiH2A (cyan), anti-γH2AFX (violet). MSCI markers were identified in the sex chromosomes (as a control experiment to confirm the distribution of proteins in multiple sequential staining assays). (**A–D**) ATR is localized in chromatin of the asynaptic zone of the XX bivalent. (**E–H)** Nlb is SUMO-1 and DAPI positive (grey). (**I–L**) ubiH2A is located within the entire sex bivalent. The sex bivalent was open, telomeric synapsis was absent, and centromeric signals occurred at the axial element. (**M–P)** The sex bivalent "twisted" into a tangle, and was surrounded by a cloud of histone γH2AFX. The arrows indicate Nlbs.


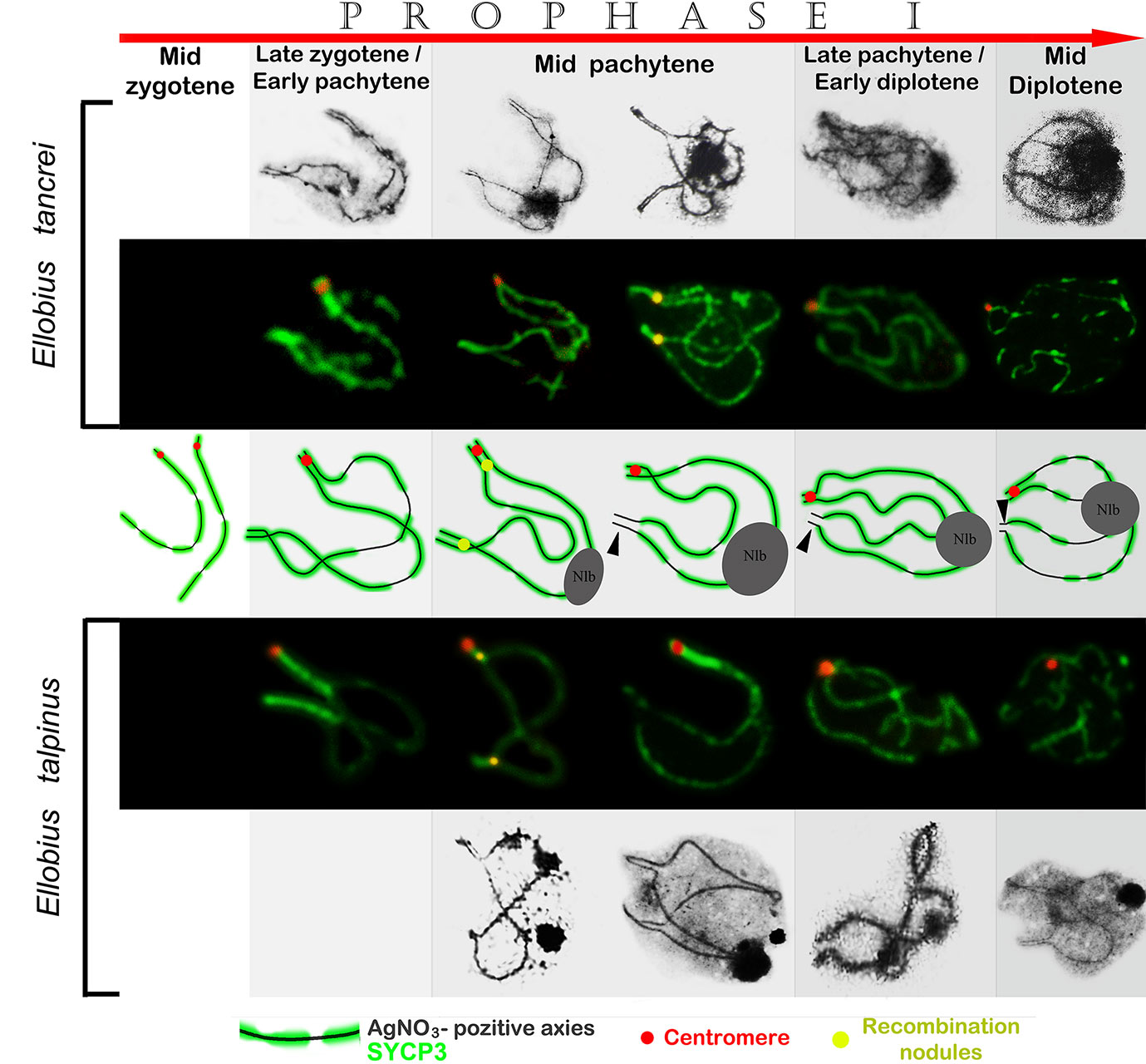


Figure S2: The progression of sex bivalents at meiotic prophase I in two mole vole species.


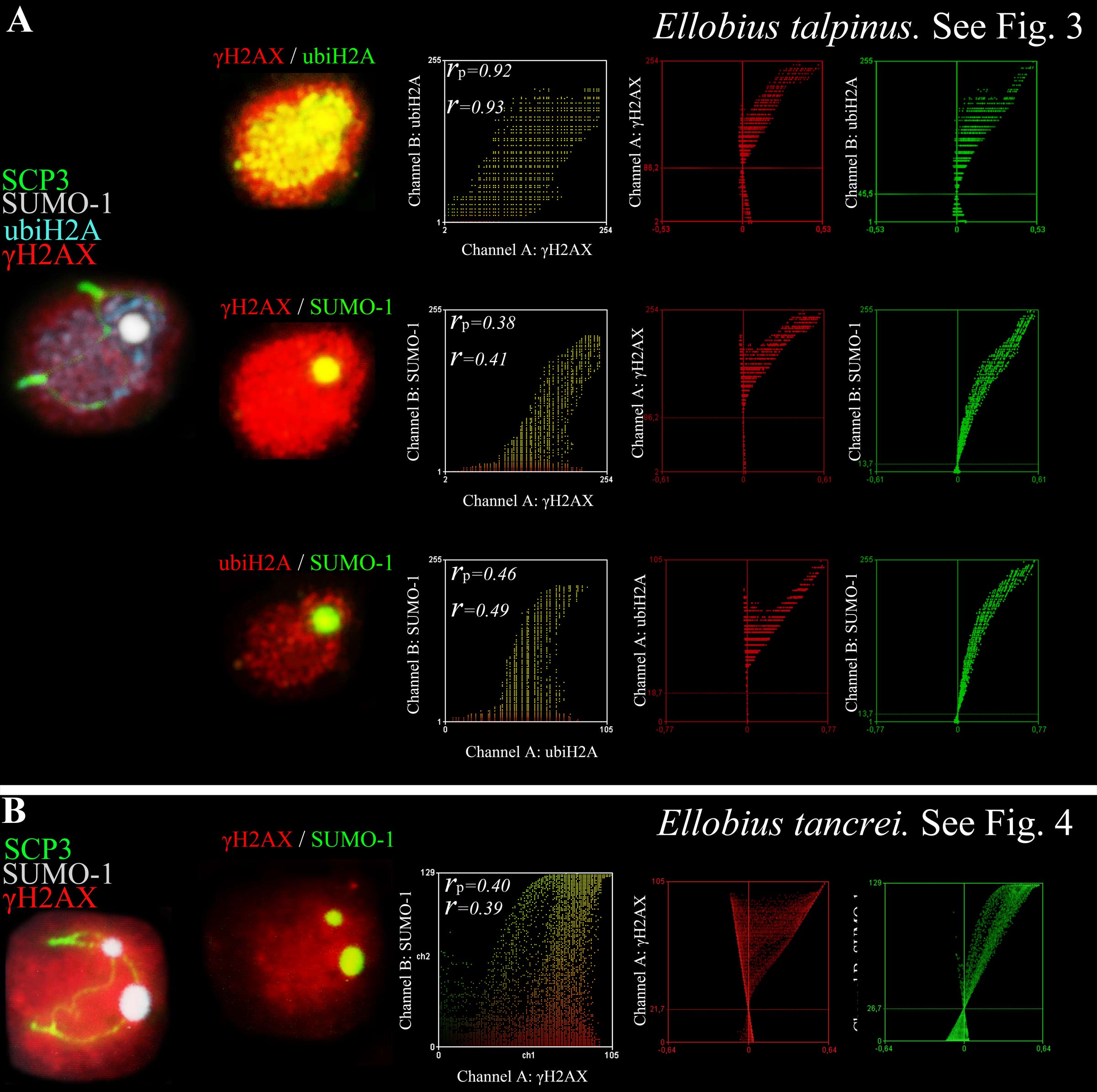


Figure S3: Intensity correlation analysis (ICA) represented by scatter plots of the paired intensities of the two channels (γH2AFX - ubiH2A; γH2AFX - SUMO-1; ubiH2A – SUMO-1) and the individual fluorescence intensities of channel A (red) and channel B (green) (see Fig. 3). The Pearson correlation coefficient *r*_p_ and overlap correlation coefficient *r* are indicated on the scatter plots.


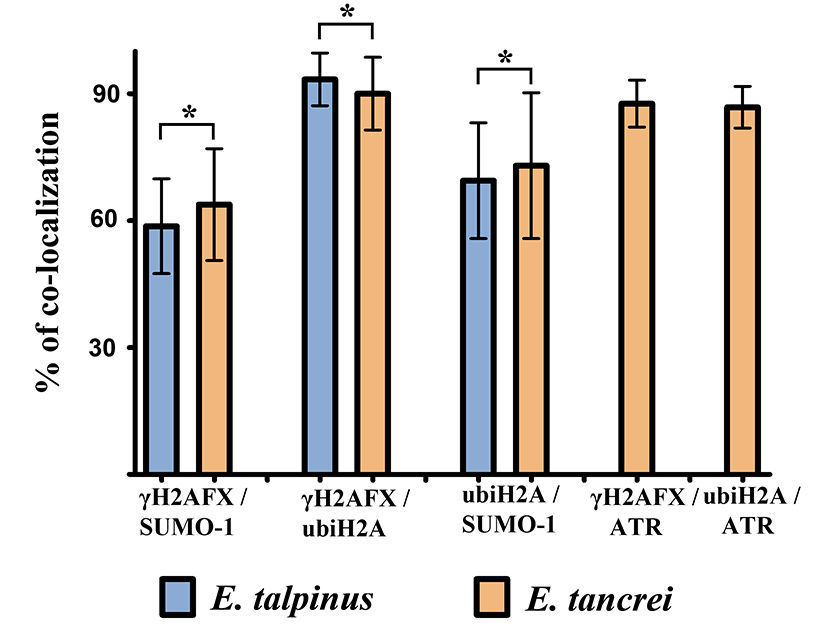


Figure S4: Degree of co-localization for signals in sex bivalents of two mole voles species. At the *y*-axis, a percentage of co-localisation signals according to overlap correlation coefficients (*r*) is shown. There are no significant differences in co-localization for γH2AFX/SUMO-1, ubiH2A/SUMO-1, and γH2AFX/ubiH2A (* *P*>0,05).
